# An inhibitor of RORγ for chronic pulmonary obstructive disease treatment

**DOI:** 10.1038/s41598-022-12251-z

**Published:** 2022-05-24

**Authors:** Harshada Desai, Megha Marathe, Varada Potdar, Prabhakar Tiwari, Ashwini Joshi, Sheetal R. Kadam, Arti Rajesh Joshi, Abhay Kulkarni, Vikram Bhosale, Avinash Hadambar, Bhavik Lodhiya, Venkatesha Udupa, Dayanidhi Behera, Sachin S. Chaudhari, Sanjib Das, Malini Bajpai, Nagaraj Gowda, Pravin S. Iyer

**Affiliations:** 1 Department of Biological Research, Glenmark Pharmaceuticals Limited, Glenmark Research Centre A-607, MIDC Mahape, Navi, Mumbai 400709 India; 2 Department of Toxicology, Glenmark Pharmaceuticals Limited, Glenmark Research Centre A-607, MIDC Mahape, Navi, Mumbai 400709 India; 3 Department of Drug Metabolism and Pharmacokinetics, Glenmark Pharmaceuticals Limited, Glenmark Research Centre A-607, MIDC Mahape, Navi, Mumbai 400709 India; 4 Department of Chemical Research, Glenmark Pharmaceuticals Limited, Glenmark Research Centre A-607, MIDC Mahape, Navi, Mumbai 400709 India; 5 Head NCE Research, Glenmark Pharmaceuticals Limited, Glenmark Research Centre A-607, MIDC Mahape, Navi, Mumbai 400709 India

**Keywords:** Biochemistry, Cell biology, Drug discovery, Immunology, Diseases

## Abstract

The role of RORγ as a transcription factor for Th17 cell differentiation and thereby regulation of IL-17 levels is well known. Increased RORγ expression along with IL-17A levels was observed in animal models, immune cells and BAL fluid of COPD patients. Increased IL-17A levels in severe COPD patients are positively correlated with decreased lung functions and increased severity symptoms and emphysema, supporting an urgency to develop novel therapies modulating IL-17 or RORγ for COPD treatment. We identified a potent RORγ inhibitor, PCCR-1 using hit to lead identification followed by extensive lead optimization by structure–activity relationship. PCCR-1 resulted in RORγ inhibition with a high degree of specificity in a biochemical assay, with > 300-fold selectivity over other isoforms of ROR. Our data suggest promising potency for IL-17A inhibition in human and canine PBMCs and mouse splenocytes with no significant impact on Th1 and Th2 cytokines. In vivo, PCCR-1 exhibited significant efficacy in the acute CS model with dose-dependent inhibition of the PD biomarkers that correlated well with the drug concentration in lung and BAL fluid, demonstrating an acceptable safety profile. This inhibitor effectively inhibited IL-17A release in whole blood and BALf samples from COPD patients. Overall, we identified a selective inhibitor of RORγ to pursue further development of novel scaffolds for COPD treatment.

## Introduction

Chronic obstructive pulmonary disease (COPD) is a progressive life-threatening lung disease which imposes a considerable worldwide disease burden and is anticipated to be the fourth leading cause of morbidity and mortality in USA^1^ (American Lung Association, Research Report, GOLD 2020). COPD is characterized by persistent respiratory symptoms and airflow limitation due to the airway and/or alveolar abnormalities, usually caused by significant exposure to noxious particles or gases^1^. The airflow limitation is a result of complex mechanisms and is not limited to neutrophilic airway inflammation^2^. Present therapies for COPD primarily address symptoms rather than modifying the disease. The mainstay of current therapies includes either short- and long-acting inhaled bronchodilator therapies or combinations thereof, anti-inflammatory therapies like inhaled corticosteroids (ICSs), phosphodiesterase-4 inhibitors and azithromycin, and other therapies such as methylxanthines, mucolytic agents, oxygen supplementation and surgical intervention^3^.

The literature suggests anti-inflammatory inhibitors targeting various targets such as PDE4, p38 MAPK, and PI3K inhibitors, have been explored for COPD with variable success^4,5^. Among these, only a PDE4 inhibitor has been proven in large Phase III clinical trials to be efficacious with both bronchodilatory and anti-inflammatory properties [NCT02986321]^6^. Unfortunately, its benefit is often eclipsed by an adverse effect profile of gastrointestinal symptoms that significantly impacts quality of life^6,7^ and thereby limits its use in patients with advanced stages of COPD^8–10^. Another class of anti-inflammatory drugs, corticosteroids also showed limitations with their resistance towards neutrophilic lung inflammation in COPD^11,12^. These limitations underscore a global urgency to develop new anti-inflammatory drugs with disease modifying ability with a satisfactory safety profile. Considering this lacuna in anti-inflammatory therapies for COPD, RORγt inhibition shows promise, but still needs to be explored. Based on clinical trial results of a safe and well tolerated antagonist of RORγ, VTP-43742 showed efficacy in patients with psoriasis in a phase II study (NCT02555709) indicating its anti-inflammatory role. Similarly, oral JNJ‑61803534SHR168442^13^ inhibitor and the topical application of SHR168442^14^ for psoriasis are additional examples of the anti-inflammatory benefit of RORγ inhibition. Thus it is hypothesized that a RORγ antagonist could be a novel therapeutic target for COPD by regulating IL-17 mediated inflammation. Furthermore, literature reports on experimental animals have shown treatments with anti-IL-17 contributed to improvement of most parameters of inflammation and extracellular matrix remodeling in the model of eNO synthase of lung injury^15^.

The role of T lymphocytes, neutrophils and alveolar macrophages in COPD has been well established, characterized by the production of IL-17A, IL-17F, IL-23 and IL-22 cytokines^14^. The transcription factor, RORγ, is known to be necessary for Th17 cell differentiation and production of IL-17 in autoimmune disorders^17–20^ However, the precise role of IL-17 producing cells in COPD is not well elucidated. Of particular note, is the observation, that in animal models of cigarette smoke-induced emphysema, a significantly higher number of IL-17 and IFN-γ producing cells with an increased mRNA expression of RORγ and Th17 dependent cytokines were observed^16,18,20^. Mice exposed to cigarette smoke (CS) and treated with anti-IL-17 antibody (Ab) (acute 4 days model and chronic 4-month model) have shown significant inhibition of the total cells and neutrophils in bronchoalveolar lavage fluid (BALf), along with an improvement in the lung pathology induced by chronic CS exposure^22,23^. In COPD patients, the expression of IL-17 and IL-22 were increased in both the bronchial mucosa and lung tissue compared to healthy subjects^24^ Also, an increased level of IL-17A was observed with increasing GOLD stage severity and during exacerbations^19,25^, in induced sputum and lung biopsy tissue of COPD patients^26,27^. Similarly, an increase in the RORγ expression was also seen in COPD patients along with an increase in the IL-17^+^ cell-related chemokine receptors CCR6 and IL-23R in at least 10–20% of patients^28^, providing further evidence for a potential significant role of RORγ as a therapeutic target in COPD.

In this study, we aimed to identify a selective RORγ inhibitor demonstrating a potent anti-inflammatory effect in respiratory disease endotypes characterized by RORγ up-regulation. We have evaluated the potency of a novel molecule PCCR-1 inhibiting RORγ and its selectivity over other ROR isoforms in a biochemical assay. The inhibition by PCCR-1 through RORγ was evaluated using transactivation assay as well as by modulation of RORγ expression in parallel to IL-17 expression in TCR differentiated PBMCs. The inhibitory effect of PCCR-1 was estimated in a cellular mechanistic study for IL-17A inhibition across multiple species (human and canine PBMCs as well as in mouse splenocytes) under TCR and Th17 differentiating conditions, along with assessment of its effect on Th1 and Th2 dependent cytokines. In addition, we have evaluated the effect of RORγ inhibition in cells from lung pleural fluid by measuring the levels of IL-17 in the macrophages and neutrophils across species. In addition to establish clinical relevance of our observations from the animal model, we evaluated the effect of PCCR-1 on IL-17 levels in whole blood and banked samples of BALf from COPD patients and also evaluated its PK-PD correlation. PCCR-1 was found to selectively abrogate the Th17 dependent cytokines across species and has the essential properties of a drug to be evaluated in COPD patients.

## Results

### PCCR-1 inhibits RORγ activity and modulate cellular function

**Figure 1:**
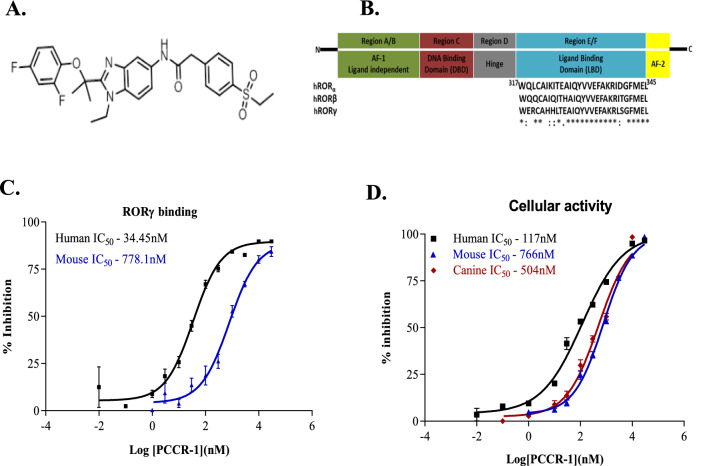
PCCR-1 inhibits RORγ both biochemical and cellular across species. Biochemical and cellular activity of RORγ inhibitor. (**A**) Chemical structure of PCCR-1. (**B**) Functional domains of ROR. Schematic diagram of domain structure of RORs with a N-terminal ligand-independent activation function 1 (AF-1) domain, followed by a DNA binding domain (DBD), a hinge domain, and a ligand-binding domain with an activation function 2 (AF-2) domain. Sequence alignment of the ligand binding domain of human RORα, RORβ, and RORγ performed using^56^ methodology. Identical and partially conserved residues are labeled with an asterisk and colon respectively. (**C**) Biochemical activity. Effect on cofactor recruitment to the human (n = 15) and mouse (n = 6) RORγ LBD measured by TR-FRET assay. (**D**) Cellular activity. Dose dependent inhibition of IL-17 production from human PBMC (n = 24), mouse splenocytes (n = 13) and canine PBMC (n = 6). ‘n’ is the representative of number of experimental replicates. (The human, mouse and canine basal values were in the range of 58.41 pg/ml, 9.24 pg/ml and 1.5 pg/ml, on induction the IL-17 values were in the range of 1600 pg/ml, 746 pg/ml and 1503 pg/ml respectively).

To identify small molecule RORγ inhibitors, we conducted in vitro screening using a TR-FRET binding assay to determine the inhibition of RORγ with a series of different compounds. With extensive effort and from hit identification to lead optimization, PCCR-1 was identified^29^ (Fig. 1A). Based on the homology of the ligand binding domains (LBD) between different ROR isoforms (hRORα, hRORβ and hRORγ), slight variations have been observed in the LBD regions which possibly lead to differences in competitive binding between the isoforms (Fig. 1B). Simultaneous binding of a tracer and antibody results in an increase in fluorescence resonance energy transfer (FRET), which decreases with displacement of the tracer upon inhibitor binding. The IC_50_ of PCCR-1 was estimated using a specific LBD of human and mouse RORγ and showed an inhibition potency of 34.45 nM and 778.1 nM respectively (Fig. 1C). PCCR-1 showed specific inhibition of human RORγt in a transactivation assay with a potency of 111 nM (Supplementary Fig. 2D) which is comparable to the potency of cellular inhibition of IL-17 release (Fig. 1C).

Inhibition of RORγ by PCCR-1 was also estimated in a functional assay by measuring the inhibition of Th17 dependent cytokine IL-17A release in human peripheral blood mononuclear cells (PBMCs), mouse splenocytes and canine PBMC stimulated with their respective anti-CD3 and anti-CD28 antibodies. PCCR-1 inhibited IL-17A release in human PBMC, mouse splenocytes and canine PBMC with a potency of 117 nM, 766 nM and 504 nM respectively (Fig. 1D). The mouse splenocytes showed relatively lower potency for IL-17A inhibition when compared across species, including rat IL-17 (80.63 nM, Supplementary Fig. 1A). PCCR-1 also showed relatively lower potency for IL-17F levels using hPBMC (788.2 nM, Supplementary Fig. 1B). These results indicate that, PCCR-1 is a potent inhibitor of RORγ and possesses a cellular inhibitory potency for IL-17A release across species.

PCCR-1 treated PBMCs showed downregulation of RORγt and also significantly inhibited IL-17A mRNA expression across healthy human subjects. PCCR-1 reduced the IL-17 expression from human PBMC of IL-17 significantly across healthy subjects in line with RORγt suppression indicating the down-stream effect on IL-17 marker (Supplementary Fig. 1C).

PCCR-1 inhibited IL-17 release in both mouse and human peritoneal neutrophils with a potency of 245.3 nM and 3661 nM respectively (Supplementary Fig. 2A,C). PCCR-1 also showed inhibition in IL-17 at a lower potency in peritoneal macrophages, with an IC_50_ of 1390 nM (Supplementary Fig. 2B).

Therefore, in addition to Th17 cells, other cells types treated with PCCR-1 also demonstrated suppression of IL-17 release, indicating a downstream effect of RORγ inhibition. Thus irrespective of cell type, the release of Th17 cytokines could be regulated primarily by the transcriptional modulator RORγ.

### PCCR-1 binds strongly to RORγ

**Figure 2:**
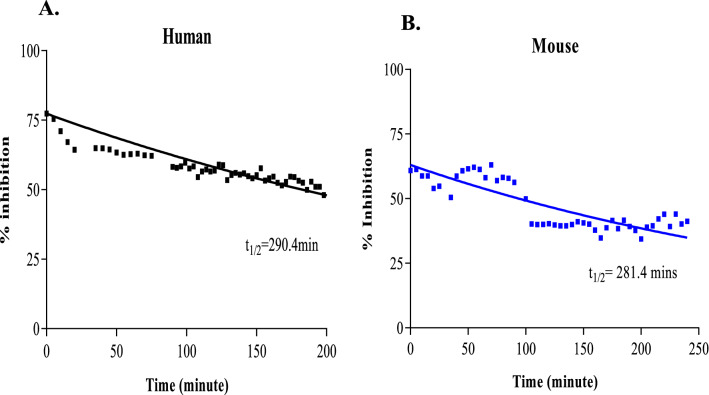
PCCR-1 strongly binds with RORγ across species. Binding affinity of PCCR-1 for human and mouse RORC/γ observed by jump dilution method. (**A**). Human binding affinity (**B**). Mouse binding affinity. The data representative of four separate experiments.

To understand the inverse agonist behavior at the molecular level, interaction with the nuclear receptor RORγ was analyzed in binding assays by estimating its dissociation constant (K_off_) and half-life (t_1/2_). The binding of a ligand stabilizes the conformation of the LBD while binding of a cofactor modifies receptor activity. Binding reversibility was evaluated by adding an excess of competing ligand in the FRET assay and evaluating the displacement of the tracer with inhibitor. Binding of the PCCR-1 with the LBD caused reduction in FRET on antibody addition forming a GST (Glutathione S-transferases) tagged RORγ-antibody complex. The dissociation assay was performed using the jump dilution method with PCCR-1 at a concentration showing maximum binding potency or IC_90_. The dissociation was tested on addition of an excess of a competitive ligand, the tracer. PCCR-1 was observed to be a strong binder showing 20–25% dissociation from the target protein with adequate target residence time for human at t_1/2_ 290 min and mouse at t_1/2_ 281.4 min RORγ proteins (Fig. 2A,B). The absolute dissociation was observed to be 50% in both species till 3 h of measurement.

### PCCR-1 selectively inhibits RORγ and no other nuclear receptors or pharmacological off-target receptors

Based on the homology of the ligand binding domains, 3 different isoforms of ROR (hRORα, hRORβ and hRORγ) are reported^30^. Small variations in the LBD regions possibly lead to differences in competitive binding between the nuclear receptors of the ROR isoforms (Fig. 1b). Similar to the assessment of RORγ binding assay, TR-FRET binding assays were also used to assess the affinity of PCCR-1 for other ROR isoforms. PCCR-1 showed IC_50_ of > 10 µM (> 313.7 fold) for the rest of the non RORγ isoforms as well as for other major nuclear receptors (Table 1). This was reconfirmed in cellular functional assays for the selected isoforms and nuclear receptors using Gal4 DBD with luciferase reporter system from Indigo (PA, USA). PCCR-1 was found to have no effect in the transactivation assays done to show RORα inhibition (Indigo, Table 1). In addition, both cellular agonist and antagonist effects as well as for enzyme activity were evaluated with a known reference compound for each target, using a radiolabeled method (Eurofins Cerep, France, Table 1). PCCR-1 showed no significant effect (> 1000-fold selectivity) against other pharmacological target receptors, ion channels and enzymes as compared to the reference compounds. Hence results indicate that PCCR-1 is a selective inhibitor of RORγ that specifically binds to RORγ LBD domain and not to other tested enzymes or nuclear receptors.

**Table 1:** In vitro selectivity profile for PCCR-1.

Selectivity Profile for PCCR-1 : RORg IC_50_ 31.87 nM												
PCCR-1	RORα		RARα		LXRα		LXRβ		RXR		FXR	
Concentration (µM)	1	10	1	10	1	10	1	10	1	10	1	10
% Inhibition	20.91	19.82	0.00	0.00	11.38	30.37	28.11	46.66	0.00	8.24	21.45	19.81
Fold over hRORγ	> 313.7		> 313.7		> 313.7		> 313.7		> 313.7		> 313.7	

**Selectivity profile for PCCR-1: CEREP panel**												
Nuclear receptors: binding assays (RORα, RARα, LXRα, LXRβ, RXR, FXR)	NSE at 10 µM											
Nuclear receptors: functional assays(h/m): (RORα, FXR, RARγ, PPARγ, LXRα/β, RXRγ)	hFXR (antagonism): ~ 10 µM mPPARγ(agonism): V wk											
Kinases (~ 40 in no)	NSE at 10 µM											
Cerep Panel of receptors (binding/functional assays)	NSE at 10 µM except#											
IC50/% inhibition at 10 µM	#Alpha 1A (antg): 190 nM, Alpha 1D: 550 nM, GABA Cl: 4.8 µM, Ca Channel (L type): 2.6 µM, NK3: 56%											
hERG (IC50)	6.0 µM (moderate)											

*NSE* No significant effect. *V* *wk* very weak (very low-level agonist activity seen with mPPARγ). We represented the Selectivity Profile of PCCR-1: Biochemical activity of PCCR-1 and fold selectivity measured across nuclear receptors w.r.t RORγ and Binding/ functional selectivity profile against panel of receptors and enzymes from Eurofins Cerep, France.

### PCCR-1 selectively inhibits IL-17 and not Th1 and Th2 cytokines

**Figure 3:**
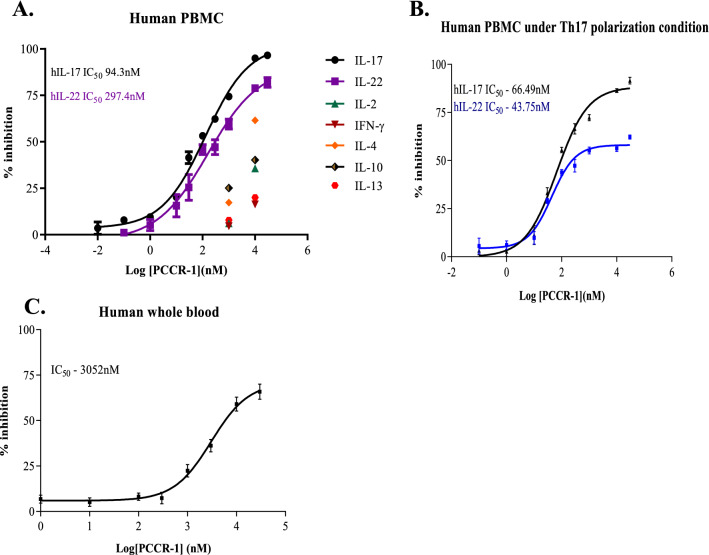
PCCR-1 strongly binds with RORγ across species under Th17 polarization condition and also modulate in whole blood. Th17 dependent regulation of PCCR-1: Dose dependent inhibition of (**A**) Th1, Th2, Th17 cytokine production from human PBMC, (**B**) Th17 cytokine production from human PBMC under Th17 polarization conditions (n = 2), the basal value was negligible and on stimulation the IL-17 level was in the range of 1100-1500 pg/ml across donors and 17-45 pg/ml in basal and on stimulation the IL-22 was in the range of 500-835 pg/ml (**C**) IL-17 cytokine from human whole blood (n = 10) with negligible basal and IL-17 in the range of 267—1532 pg/ml. ‘n’ is a representative data from two to three donors in replicates.

An increase in the levels of Th17 dependent cytokine in the peripheral blood of COPD patients as compared to the Th1 and Th2 dependent cytokines has been well documented in a subset of COPD patients^24^. A comparative study was performed under TCR activating conditions to evaluate the potencies of PCCR-1 for inhibition across Th1, Th2 and Th17 dependent cytokines using hPBMCs. PCCR-1 showed an IC_50_ of 94.3 and 297.4 nM for IL-17A and IL-22, respectively. However, PCCR-1 did not show significant inhibition of Th1 (IL-2 and IFN-γ) and Th2 (IL-4, IL-13 and IL-10) dependent cytokines even at 10 µM (Fig. 3A). Similarly, PCCR-1 potency was evaluated under Th17 differentiation conditions for IL-17A and IL-22 release and we observed comparable IC_50_ potencies of 66.49 nM and 43.75 nM, respectively. However, a ceiling effect for IL-22 release inhibition was observed at 60% (Fig. 3B). Also, PCCR-1 showed IL-17A inhibition in human whole blood under TCR stimulatory conditions with an IC_50_ of 3052 nM (Fig. 3C). These results indicate that PCCR-1 is a potent and selective RORγ inverse agonist that specifically inhibits Th17 dependent cytokines under TCR and Th17 dependent stimulatory conditions over Th1 and Th2 cytokines.

### PCCR-1 potently inhibits IL-17A in CS mouse model and COPD patient’s samples

**Figure 4:**
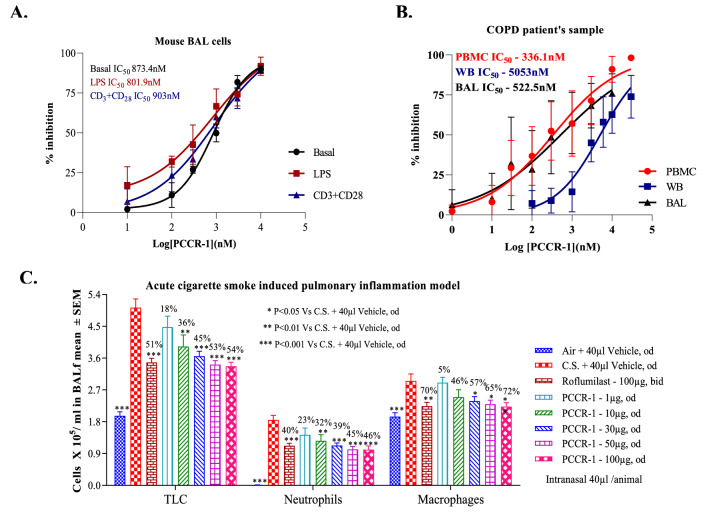
PCCR-1 inhibits ex vivo release of IL-17 in cultured BAL cells from Cigarette Smoke exposed animals and also modulate in COPD patients in PBMC and BALf cells. Ex vivo and in vivo studies of PCCR-1. (**A**) Dose dependent inhibition of IL-17 cytokine from cultured BAL cells from cigarette smoke exposed mice in basal and induced conditions (**B**) Dose dependent inhibition of IL-17 cytokine from human whole blood (n = 5), BAL cells (n = 10) and PBMC (n = 16) derived from COPD patients. The basal value was negligible and on stimulation the IL-17 level was in the range of 49.15, 40.9 and 2098 pg/ml for Whole blood, BAL and PBMC across donors (**C**) Effect on Acute Cigarette Smoke Induced Pulmonary Inflammation mice model at different doses of PCCR-1 (n = 7 per group). ‘n’ is a representative data from one to four donors in replicates.

The effect of PCCR-1 was evaluated ex vivo using BALf cells from an acute cigarette smoke induced animal model. Animals were exposed to cigarette smoke for 7 days and BALf cells were collected and treated with different doses of PCCR-1 under various stimulatory conditions. IL-17A levels and PCCR-1 inhibitory potentials were compared in basal and LPS or anti-CD3 + anti-CD28 antibody induced conditions and found to be comparable (Fig. 4A), suggesting that RORγ inhibition potentially suppressed inflammation across various inflammatory stimuli. Stimulation by LPS may represent the inflammatory state during a COPD exacerbation, supporting a potential benefit of PCCR-1 in this disease state.

A selective up-regulation along with a prominent role of the IL-17A isoform as compared to IL-17F is well documented in the bronchial submucosa and infiltrating inflammatory cells of the small airways of COPD patients^21^ (Zhang et al., 2013). In this study, BALf cells, PBMCs and whole blood were evaluated from COPD patients. PCCR-1 showed a concentration dependent inhibition of TCR stimulated IL-17 release from COPD lung BALf cells, PBMCs and whole blood, with potencies of 522.5 nM, 336.1 nM and 5053 nM respectively (Fig. 4B). These results demonstrate a good translation of PCCR-1 activity in COPD patients and also in a mouse CS model.

### PCCR-1 shows significant efficacy in acute cigarette smoke model

Cigarette smoke exposure for 7 days caused significant increase in the total cell count in BAL fluid, including leukocytes, neutrophils and macrophages as compared to an air exposed control group. PCCR-1 was dosed once a day for 7 days through the intranasal route and showed a dose dependent inhibition of total leukocytes between 1 µg and 50 µg/animal, with inhibition of total leukocytes at 50 µg, similar to that observed with 100 µg BID of Roflumilast (used as a positive control). PCCR-1 at 10, 30, 50 and 100 µg per mouse also significantly obtunded cigarette smoke mediated increase in neutrophil and macrophage counts in BAL fluid (Fig. 4C).

### PCCR-1 shows high lung concentrations in mice after intranasal administration

A single intranasal dose resulted in high lung exposures of PCCR-1 in mice, as suggested by a mean lung to plasma Cmax and AUClast ratios that were found to be > 1000 at both the doses (20 and 50 µg), respectively (Table 2). Peak lung concentrations at both the doses were observed at 2 to 4 h post dose. Quantifiable levels in lungs were observed up to 24 h post dose. The mean apparent elimination half-life and AUC∞ were not estimated as the data point describing elimination phase were inadequate to get acceptable accuracy in these parameters.

**Table 2:** Concentrations of PCCR-1 observed in mice exposed to cigarette smoke at 1 µg, 10 µg, 30 µg, 50 µg and 100 µg on day-8 after intranasal dose of PCCR-1.

Dose (µg/animal)	Total concentration (nM)		
	Plasma	Lung	BAL fluid
1	< 1.85	5476.89	25.74
10	< 1.85	53,604.44	159.82
30	20.64	171,018.48	389.59
50	33.18	379,414.05	778.96
100	84.70	607,682.07	1320.08

Further, after the 7-day intranasal treatment with PCCR-1 in the acute CS-model in mice, the results observed from the PD study were in line with the PK data, wherein very high concentrations (> 1000-fold) of PCCR-1 were observed in lung as compared to the plasma across all the tested doses (1 to 100 µg). A very good correlation of the lung and BAL fluid concentrations of the compound versus inhibition of the PD biomarkers (total leukocyte count, neutrophils and macrophages) were observed with good dose-dependent inhibition profiles (Supplementary Table 1, Supplementary Fig. 3). Overall, the data indicated a strong PK-PD correlation with the compound.

### PCCR-1 demonstrates an acceptable toxicology profile

Rats were exposed to PCCR-1 aerosols at 3.4, 10.8 and 32.1 mg/kg/day (delivered dose) for 60 min daily for 14 days using a snout only exposure technique via a modular stainless steel flow past inhalation chamber at indicated doses as mentioned in Table 3. No treatment-related findings noted, including myeloid:erythroid (M:E) ratios in any dose levels except minimal changes in the reticulocytes and adrenal glands.

**Table 3:** Summary of Toxicokinetic Parameters (blood) for PCCR-1 in Sprague Dawley rats with inhalation administration.

Delivered Dose (mg/kg/day)	Day	Sex	C_max_ (ng/mL)	C_max_/Dn (ng/ml/mg/kg)	T_max_ (hr)	AUC_0-tlast_ (ng.hr/mL)	AUC_0-tlast_/Dn (ng.hr/mL/mg/kg)	t_1/2_ (hr)	t_last_ (hr)
3.4	1	Female	201.6 (31.9)	59.3	3.0	1002.1 (161.9)	294.7	−	9.0
		Male	176.7 (4.1)	52.0	3.0	962.8 (97.3)	283.2	–	9.0
		**Mean**	**189.1 (18.0)**	55.6	**3.0**	**982.4 (129.6)**	**289.0**	–	**9.0**
3.4	14	Female	240.9 (38.8)	70.9	5.0	2612.6 (204.5)	768.4	–	25.0
		Male	189.9 (43.5)	55.9	3.0	2503.9 (231.2)	736.4	6.4	25.0
		**Mean**	**215.4 (41.2)**	63.4	**4.0**	**2558.2 (217.9)**	**752.4**	**6.4**	**25.0**
10.8	1	Female	543.6 (120.4)	50.3	3.0	7269.2 (1555.2)	673.1	5.8	25.0
		Male	370.1 (28.7)	34.3	3.0	2913.3 (215.4)	269.7	6.0	25.0
		**Mean**	**456.9 (74.5)**	42.3	**3.0**	**5091.2 (885.3)**	**471.4**	**5.9**	**25.0**
10.8	14	Female	679.1 (120.5)	62.9	3.0	8054.8 (830.9)	745.8	5.1	25.0
		Male	358.7 (50.9)	33.2	5.0	5436.1 (358.1)	503.3	–	25.0
		**Mean**	**518.9 (85.7)**	48.0	**4.0**	**6745.4 (594.5)**	**624.6**	**5.1**	**25.0**
32.1	1	Female	935.9 (178.9)	29.2	3.0	9470.7 (807.7)	295.0	6.9	25.0
		Male	533.5 (90.7)	16.6	3.0	5406.1 (757.5)	168.4	6.8	25.0
		**Mean**	**734.7 (134.8)**	22.9	**3.0**	**7438.4 (782.6)**	**231.7**	**6.8**	**25.0**
32.1	14	Female	1148.7 (192.9)	22.9	5.0	16,183.6 (1943.9)	504.2	–	25.0
		Male	885.4 (174.8)	27.6	3.0	8233.6 (1353.7)	256.5	–	25.0
		**Mean**	**1017.0 (183.8)**	31.7	**4.0**	**12,208.6 (1648.8)**	**380.3**	**–**	**25.0**

Mean values are in bold.

*: T_max_ and T_last_ are calculated from the start of Inhalation dosing; Inhalation dosing duration was 1.0 h; C_max_/Dn and AUC_0-tlast_/Dn are C_max_ and AUC_0-tlast_ normalized to delivered dose (mg/kg). Mean (SE) is provided for C_max_ and AUC_0-tlast_ and mean for other PK parameters. *Dn* delivered dose.

The TK assessment was done by measuring the concentrations of PCCR-1 in blood. The C_max_ and AUC on both Day 1 and Day 14 appeared to be comparable between male and female rats at the low dose level (Table 3). However, at the mid and high dose levels, the C_max_ and AUC in females were approximately 1.3–1.9 fold and 1.5–2.5 fold higher than males, respectively. The blood concentration profiles indicated that in general, the C_max_ and AUC on both day-1 and 14 increased with an increase in dose level. With once-daily inhalation administration for 14 days, PCCR-1 AUC in blood accumulated about 1.5–2.6-fold relative to that of day-1, except for female rats at 10.8 mg/kg/day dose level where there was no considerable accumulation observed.

A slightly increased reticulocyte count was noted in the mid and high dose group females (1.27 and 1.44 fold compared with controls) and was statistically significant in the high dose group compared with controls (Table 3). A marginal increase in reticulocyte count was also noted in the high dose males compared with the control group (1.09-fold). Statistical significance noted in other haematological parameters was considered incidental. Although statistically not significant, slightly higher group mean adrenal weights (absolute and relative to body weight/brain weight) were observed in the mid and high-dose females when compared with the control group (Table 4). No treatment-related histopathological changes were noticed in any organs, including the respiratory system, except the adrenal gland that showed bilateral diffuse hypertrophy of the zona fasciculata in the mid and high-dose females. This correlated with high adrenal gland weights. Based on these results, the maximum tolerated dose of PCCR-1 was considered to be 32.1 mg/kg/day.

**Table 4:** Group mean reticulocyte count, adrenal gland weight and microscopic changes after dosing of PCCR-1.

Group	Males (n = 5/group)				Females (n = 5/group)			
	1	2	3	4	1	2	3	4
Delivered dose (mg/kg/day)	0	3.4	10.8	32.1	0	3.4	10.8	32.1
Hematology (mean ± SD)								
Reticulocyte count (10^9^/L)	263 ± 40.8	207.8 ± 37.5	250.5 ± 89	287.7 ± 62.6	205.1 ± 43.9	230.3 ± 40.7	260.5 ± 29.6	295.4 ± 40.3*
**Weight changes (mean ± SD)**								
Absolute weight (g)	0.0635 ± 0.0051	0.0602 ± 0.0072	0.0650 ± 0.0076	0.0581 ± 0.0083	0.0790 ± 0.0082	0.0864 ± 0.0141	0.0929 ± 0.0111	0.0950 ± 0.0139
Relative to body weight (%)	0.01689 ± 0.00143	0.01618 ± 0.00252	0.01748 ± 0.00160	0.01553 ± 0.00171	0.02744 ± 0.00323	0.02898 ± 0.00423	0.03326 ± 0.00448	0.03168 ± 0.00549
Relative to brain weight (%)	3.04418 ± 0.30705	2.79962 ± 0.30163	3.12413 ± 0.33757	2.80336 ± 0.30407	3.87961 ± 0.47563	4.14160 ± 0.65041	4.56772 ± 0.55188	4.65696 ± 0.61762
**Microscopic changes**								
Hypertrophy; zona fasciculata, diffuse	0	–	–	0	0	0	2	3
Minimal	0	–	–	0	0	0	0	1
Mild	0	–	–	0	0	0	2	2

*P ≤ 0.01.

## Discussion

**Figure 5:**
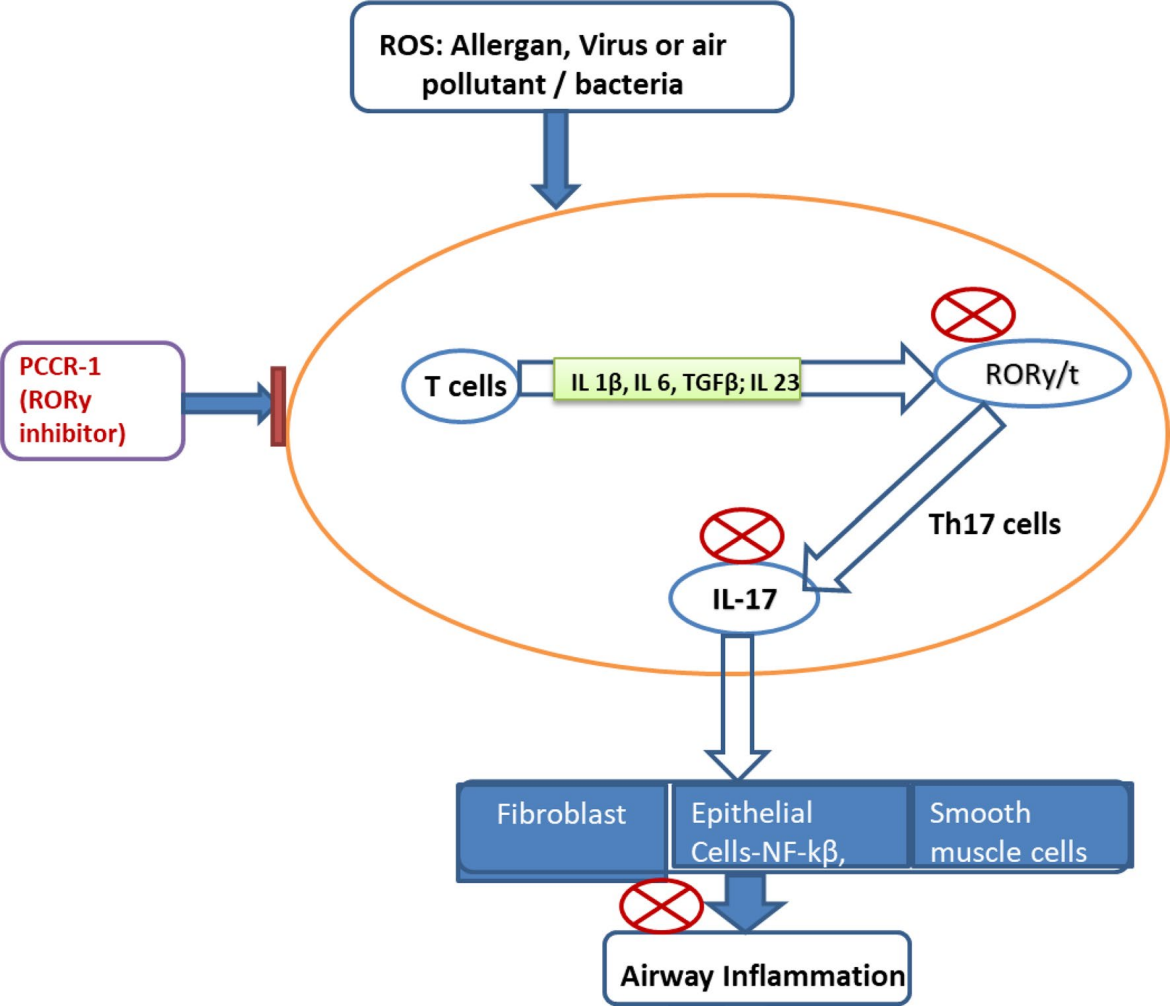
Inhibitory mechanism of PCCR-1 in RORγ and IL-17 dependent pathway. RORγ as an anti-inflammatory therapeutic target for COPD in regulating the Th17 dependent signaling. Small molecule inhibitors of RORγ leads to blockage of IL-17 release leading to prevention of various inflammatory mediators from airway epithelial cells and airway smooth muscle cells thus preventing further damage to the lung.

Th17 cytokines may play a significant role in COPD pathogenesis^31^. The presence of Th17 cells along with the release of various cytokines are predictive of disease severity and airflow limitation^27^. In addition to Th17 cells many other cells have also been shown to release these cytokines including innate lymphocytes, neutrophils and macrophages^18,32,33^. Irrespective of the cell type, the release of Th17 dependent cytokines are regulated primarily by the transcriptional modulator RORγ. Also it has been well documented that the Th17 dependent cytokines are differentially expressed across cell types and are up-regulated based on the disease type and condition^34^. Although there are some reports correlating the pharmacological regulation of Th17 dependent cytokines and their implication in COPD, this is the first report of an RORγ inhibitor as a potential therapeutic agent for COPD as depicted in the Fig. 5^35–39^.

ROR has a typical nuclear receptor domain structure consisting of four major functional domains: An N-terminal (A/B) domain followed by a highly conserved DNA-binding domain (DBD), a hinge domain, and a C-terminal ligand-binding domain (LBD). The LBDs of nuclear receptors are multifunctional and play a role in ligand binding, nuclear localization, receptor dimerization, and provide an interface for the interaction with co-activators and co-repressors^30^. Therefore, any modulations in the LBD region with a small molecule have the potential to affect the functions of nuclear receptors and influence the downstream signaling in their respective pathways. Modulation of the LBD region of RORγ by an inhibitor is demonstrated in this article and being homologous across species, this inhibition is expected to show and translate across species. PCCR-1 was profiled against a panel of receptors and other isoforms of nuclear receptors and was found to selectively inhibit RORγ, showing minimum off target effects. PCCR-1 showed specific inhibition of the human RORγt in a transactivation assay at a potency that was comparable to the cellular inhibition of IL-17 release. The expression pattern of RORγt also showed a decreasing trend on treatment with PCCR-1 along with significant reduction in IL-17 expression in the human PBMC.

Stimulated Th17 cells release various cytokines, including IL-17A, IL-17F and IL-22 which have been implicated in various inflammatory diseases including COPD^27^. IL-17A is a pro-inflammatory cytokine that modulates airway inflammation through recruitment of inflammatory cells, including neutrophils and lymphocytes, which in turn release various chemo-attractants such as CXCLs and inflammatory mediators like TNF-α and IL-6, augmenting the inflammatory state^40^. The use of Simvastatin also showed marked suppression of IL-17A and IL-22 secretion which may contribute to decrease in IL-6 and CXCL8 production in the airways of COPD patients (Fig. 5)^26^. Statins have been well known to cause beneficial effects in COPD regarding lung function decline rates and severity of exacerbations and hospitalization. Long-term use of statins reduced inflammatory factors including CRP and IL-6, increased lung function indices including FEV1% predicted and FEV1/FVC%, and reduced the risk of AECOPD^40^. Literature reports have shown a positive correlation between CRP and IL-17A, providing some evidence of this cytokine having a role in neutrophilic inflammation, a hallmark of COPD^26^. The expression of IL-17A in the bronchial submucosa was also shown to be increased in smoking associated COPD and correlated with disease severity^41^. However, neither a change in IL-17F expression^42^, nor a significant difference was observed in its levels in COPD patients or control groups. Thus IL-17A modulation may have therapeutic benefit in COPD.

We evaluated PCCR-1 a selective RORγ inhibitor that demonstrated potent inhibition of IL-17A with lower potency for IL-17F as potential therapeutic agent in COPD.

The compound specifically modulates the Th17 pathway including both IL-17 and IL-22, having no effect on Th1 and Th2 signaling as seen in the cells stimulated under TCR stimulation.

Apart from various T-helper cells, neutrophils and macrophages are also believed to play an important role in COPD. An increase in neutrophils is observed in the respiratory secretions of COPD patients, notably during exacerbations, while macrophages are present in small airways and parenchyma and are related to disease severity^32,43^. Recent reports have showed the role of RORγt in the recruitment of macrophages during hydrocarbon oil-induced chronic inflammation^44^. Therefore, modulation of macrophage activity via inhibition of facilitator cytokines by RORγ in activated T-helper cells shows potential therapeutic promise. PCCR-1 also showed inhibition of IL-17 release in other inflammatory cells including neutrophil and macrophages of mouse and neutrophils from human whole blood. The IL-17 inhibition in neutrophils was observed to be much lower as compared to the Th17 cells. However, the concentrations of PCCR-1 required for IL-17 inhibition that may impact neutrophil and macrophage inflammation, was significant as seen from the PK-PD correlation.

A seven day in vivo acute cigarette smoke exposure model showed a dose dependent inhibition of neutrophil, lymphocyte and macrophage infiltration in BAL fluid by PCCR-1. This indicated the potential benefit of a specific inhibitor of RORγ in modulating cellular infiltration in the lungs of COPD patients in a dose proportional manner. The in vivo effect was further correlated with ex vivo studies on BAL cells of cigarette smoke exposed animals where IL-17 inhibition was observed. The ex vivo model demonstrated favorable potencies across stimulating conditions for IL-17 inhibition by PCCR-1, comparable to its human IL-17 data from the BAL cells of COPD patients. To understand the translation of in vivo animal efficacy to that of human subjects, we used COPD patient samples of banked BALf cells, PBMCs and whole blood. Our data showed equivalent potencies in both human and rodents with comparable inhibition of IL-17 in both BAL cells and whole blood. Inhaled LPS causes an acute increase in airway neutrophil numbers in healthy smokers^45^ closely resembling the acute increase in airway neutrophils that occurs during COPD exacerbations. IL-17 expression has been shown to be increased in peripheral blood cells during COPD exacerbations^46^. We demonstrated significant inhibition of IL-17 under conditions of LPS exposure in an in ex vivo cigarette smoke model, where comparable inhibition was observed at similar potency as seen in the BALf from COPD patients.

The mechanism behind the long duration of action for any localized drug relates not only to its PK profile, but also to a slow rate of dissociation (residence time) from its target protein. As in the case of human M3 muscarinic receptor agonist^47^ which shows a long duration of action (of approximately 24 h): an important feature of a drug intended to treat chronic diseases, is a prolonged efficacy^48^ affording a simple, once-daily dosage regimen resulting in improved patient compliance^49^. There are many drugs currently being evaluated in clinical trials for their ability to function similarly to LAMAs (Long-acting muscarinic antagonists) with a potential for once-daily administration in COPD patients due to the established convenience of the route and once daily dosing regimen. Thus a RORγ inhibitor administered via the inhalation route, showing a strong association with its target, affording a long duration of action would fit this ideal profile of a good candidate drug for COPD. The additional advantage of a long acting RORγ inhibitor drug would be its ease of combining with currently established COPD drugs administered in once or twice daily regimen. The kinetic binding data of PCCR-1 clearly indicates strong binding to its target protein and a long dissociation from its target protein at t_1/2_ of 280–290 min (~ 50% absolute value). Literature reports indicate the long acting muscarinic antagonists showed strong binding with dissociation half-lives varying from tiotropium with t_1⁄2_ of 27 h to glycopyrrolate with t_1⁄2_ of 6.1 h^50^. PCCR-1 demonstrated a t1/2 of > 5 h with a very slow K_off_, showing only 50% dissociation after 290 min (~ 5 h) supporting a potential for twice daily dosing regimen akin to glycopyrrolate. Based on the efficacy of the molecule in the in vivo model via the intra nasal route and the known toxicities associated with the target inhibition described in the literature, the localized application of this compound was the way forward to understand its overall profile.

The role of RORα as a novel contributor of structure and function of adrenal cortex is well known and its inactivation in both sexes of mice have shown to cause structural disorganization of the adrenal cortex with increased adrenal cortex size in female mice and increased cell proliferation in males^51^. Even though PCCR-1 showed a clean profile for RORα, administration of PCCR-1 showed slight increase in reticulocyte count in the mid and high dose group females without any changes in other hematological parameters. In females, a minimal increase in the adrenal gland weight and diffused bilateral hypertrophy was observed with mid and high dose groups. This may be due to increased stimulation by adrenocorticotropic hormone (ACTH) from the pituitary gland by stress or due to adrenocortical insufficiency as a result of this effect^52,53^. The cause of the hypertrophy of the adrenal cortex in the current study was not apparent ^54,55^ and both stress and adrenocortical insufficiency were ruled out due to the lack of thymic atrophy, and hematological findings (such as reduced numbers of blood lymphocytes and/or increased numbers of blood neutrophils). The adrenal cortical hypertrophy occurred only in female rats and was not seen in any of the treated male rats due to higher exposures seen in females. Based on these results, the maximum tolerated dose of PCCR-1 was considered to be 32.1 mg/kg/day in the rat. As the compound was primarily being explored for the inhalation route, the systemic levels were quite low to expect any of these AEs. With more lead optimization efforts, an advanced compound- PCCR-2 was identified and advanced to clinical trials. PCCR-2, has shown a favorable safety profile in all Phase 1 enabling 4-week repeat dose administration studies in both rats and dogs, and has been successfully progressed to Phase 1 human Clinical Trial evaluation in USA as a potent RORγ inhibitor for COPD via-inhalation route (Clinical trial IND 144,906).

## Materials and methods

### Materials

RPMI medium, Fetal bovine serum and Penicillin and Streptomycin (GIBCO, Invitrogen corp., Carlsbad, CA, USA); Histopaque-1077, recombinant hIL-1β, recombinant TGF-β1, IL-2 (Sigma, St. Louis,MO,USA); human anti-CD3 mAb, human ant-CD28mAb, mouse ant-CD3, mouse anti-CD28 (Biolegend San Diego, CA, USA); Canine Anti-CD3 (AbD serotec); canine anti-CD28mAb (ebiosciences, San Diego, USA); recombinant IL-23 and IL-6 and ELISA kits for human IL-17, IL-22, IFN-γ, IL-2, IL-4, IL-10, Canine IL-17, Mouse IL-17A are from R&D Systems, Minneapolis, MN, USA. ROR isoforms including RORα, FXR, LXRα, LXRβ, RXR, RARα and Fluorescein-labeled coactivator peptide and lantha screen Tb-anti GST antibody (Invitrogen. MS USA); T0901317 (Calbiochem); CD1530, HX-531 (Tocris); RO41-5253 (Biomol); Fenofibrate, Guggulsterone, Trans’ retinoic acid, 9cis Retinoic acid and GW-4064 (Sigma).

### Animal experiments

Male C57BL/6 mice aged 8–10 weeks were obtained from the animal facility of Glenmark Pharmaceuticals Ltd. All animal experiments were approved by IAEC. Animals were maintained in an individually ventilated cages (IVC) in environmentally monitored air-conditioned room maintained at a temperature of 22 ± 3 °C, relative humidity of 40–70% and 12 h light/12 h dark cycle. Corn cob (BioCobb, AT&T) was used as the bedding material. Commercial pellet diet (Altromin, Germany) and community tap water passed through a reverse osmosis system (Millipore) were given. Water was provided ad libitum throughout the study period. Food was also provided ad libitum throughout the study period.

### Methods

#### RORγt-LBD and other isoforms co-activator ligand binding assay

The human and mouse RORγt cDNA clones were obtained from OriGene (MD USA). The RORγt LBDs of human (accession no. NM_00100152; RORC-LBD region 229-497aa) and mouse (accession no. NM_011281.2; RORγt-LBD region 264-516aa) were sub-cloned between Sal I and Not I, in pGEX-4T1-modified plasmid (pGEX-4T1-modified plasmid was a generous gift from Prof. Orly Reiner, from Weizmann Institute of Science, Rehovot, Israel). The correct sequence was verified by dideoxynucleotide sequencing (outsourced to Saf Labs, place). pGEX-4T1 plasmid containing human RORC -LBD was transformed and expressed in *Escherichia coli* BL21-DE3 cell stock. Protein expression was checked by western blotting. 10 nM human/mouse RORC-LBD prepared in assay buffer (25 mM HEPES; pH 7.4 with100 mM NaCl, 5 mM DTT, 0.01% BSA, and 10% Glycerol) was incubated for 60 min at 22 °C and detection mixture of 300 nM Fluorescein-D22 coactivator peptide (Invitrogen) and 10 nM Lantha screen Tb-anti GST antibody (Invitrogen. MS USA) prepared in assay buffer were added into a 384-well white plate. The plate was then incubated for 60 min at 22 °C on shaker and kept overnight at 4 °C. The next day, plate was read on Infinite F500 reader (Magellan Tecan Switzerland). TR-FRET signal was defined as the ratio 520/495. The percent activity of each dilution was determined as the ratio of background corrected signal to the background corrected signal of wells receiving only DMSO. IC_50_ values were determined by fitting percent inhibition data in GraphPad Prism (version-5.01) software.

For selectivity, the ROR isoforms RORα, FXR, LXRα, LXRβ, RXR, RARα were incubated with compound for 60 min at 22 °C followed by detection mix containing respective agonist (All trans retinoic acid for RARα, T0901317 for LXRα, LXRβ, 9-cis Retinoic acid for RXR, GW-4064 for FXR) and coactivator peptide (FD22 for RORα, PGC1α for RXR, RARα, TRAP220 for LXRα, LXRβ, SRC2-2 for FXR) and Tb-anti GST antibody prepared in assay buffer were added into a 384-well white plate. Reading taken after overnight incubation and the data analysis done as per the protocol mentioned in RORγt-LBD and co-activator ligand binding assay.

#### Cell based assays using mouse and human cells

Human whole blood from healthy subjects were procured from Clinical Research Unit, Sanpada. Clinical Research Center Glenmark (Plot No. D, 508, Turbhe, TTC Industrial Area, MIDC, Sanpada, Navi Mumbai, Maharashtra 400705, India). PBMCs were isolated using heparinized blood with Ficoll Histopaque. Briefly, the cells were seeded in 96-well plate pre-coated with human anti-CD3 mAb (10 µg/mL), post 30 min of compound treatment cells were stimulated with human anti-CD28 mAb (2 µg/mL). After 72 h of culture, the supernatant was collected for Th1/Th2 cytokines; IFN-γ, IL-2, IL-4 and IL-10 and measured by ELISA. For Th17 polarization assay, the following cytokine cocktail was used: IL-1β (10 ng/ml), IL-23(10 ng/ml), IL-6 (50 ng/ml), TGF-β1 (3 ng/ml), IL-2 (20U/ml) and supernatants were used for measuring IL-17 and IL-22 by ELISA.

For RT-PCR from human PBMC, 5X 10^6^ PBMCs were seeded in 6well pre-coated human anti-CD3 mAb (10 µg/mL) plate, post 30 min of compound treatment cells were stimulated with human anti-CD28 mAb (2 µg/mL). After 48 h cells were pelleted, washed with PBS and total RNA was extracted using TRI reagent. 3 µg of DNase treated RNA was reverse transcribed using iScript select cDNA synthesis kit (Biorad Hercules, CA, USA) to produce single-stranded cDNA. RT-PCR was performed with an real-time thermal cycler Eppendorf Mastercycler Ep (Eppendorf AG, Hamburg, Germany) using iTaq SYBR Green Supermix with ROX kit (Biorad Hercules, CA, USA) with human IL-17A specific primers: 5’- ATCTCCACCGCAATGAGGAC-3’(sense primer) and 5'-GTGGACAATCGGGGTGACAC-3' (antisense primer) and human RORγt specific primers: 5’-AGACTCATCGCCAAAGCATC-3’ (sense primer) and 5’-TCCACATGCTGGCTACACA-3’ (antisense primer). RT-PCR reaction was carried out for 40 cycles at 95 °C for 15 s, 60 °C for 15 s and 60 °C for 40 s. Data was interpreted using 2(-∆CT) Livak method of analysis.

For whole blood assay the heparinized whole blood was diluted (1:1) with saline and added in 96-well plate pre-coated with human anti-CD3 mAb (10 µg/mL). Compound treatment was done for 30 min followed by co-stimulation with anti-CD28 mAb (2 µg/mL). After 48 h of incubation supernatant was used for IL-17 estimation by ELISA.

The human neutrophils were isolated by Ficoll-Hypaque by centrifugation of blood from healthy subjects at 700 g for 30 min, followed by erythrocyte lysis with distilled water for 30 s. Cells were washed in PBS and post 1 h treatment with PCCR-1, cells were stimulated with 50 ng/mL PMA and 2 µg/mL Ionomycin. After 18 h the supernatant was collected for IL-17 estimation by ELISA.

Splenocytes were prepared from 6–8 week old BALB/c mice and cells were seeded in 96-well plates coated with mouse anti-CD3 mAb (10 µg/mL). Cells were treated with test compounds for 30 min followed by stimulation with mouse anti- CD28 mAb (3 µg/mL) for 72 h at 37 °C in 5% CO incubator. The supernatant was used for estimating mouse IL-17 by ELISA.

The peritoneal neutrophils or macrophages were isolated from C57BL/6 male mice or BALB/c female mice (6–8 wks; 18–22 g) injected with 3% or 4% thioglycollate broth. After 4 h or 4 days, RPMI medium was injected into the peritoneal cavity of mouse and peritoneal lavage was collected. The lavage was pooled and spun at 800 rpm for 10 min. The pellet was re-suspended in 1X Gey’s solution for 5 min followed by PBS wash. In case of neutrophils, the isolated cells were seeded in 48 well plate for 1.5 h. After 1.5 h, the supernatant containing peritoneal neutrophils were collected and centrifuged at 2000 rpm for 10 min and the pellet were re-suspended in 2% RPMI. In case of isolated peritoneal macrophages, the cells were seeded in 48 well plate for 3–4 h followed by PBS wash. Both the neutrophils and macrophages were treated with PCCR-1 in 2% RPMI for 1 h followed by stimulation with 10 µg/ml or 5 µg/ml of LPS for 18 h or 24 h. The supernatant collected for IL-17 estimation by ELISA.

Canine PBMCs were procured from Lonza (Cologue, Germany). Frozen canine PBMCs was quickly thawed in a 37 °C water bath and cells were seeded in 96-well plates coated with canine anti-CD3mAb (30 µg/mL). Cells were further treated with the compound similar to human PBMC with co-stimulation of canine anti-CD28 mAb (10 µg/mL). After 72 h of incubation the supernatant was collected for estimating canine IL-17 by ELISA.

For RORg transactivation assay the HEK293 cells were seeded in 100 mm dish and transfected with 4.5 µg total DNA (2.25 µg of pGL4.31 Luc2/GAL4-UAS hygro and 2.25 µg Gal4-DBD human RORγt hinge LBD/pcDNA 3.1 and 0.112 µg of pRL-CMV using Effectene transfection reagent (Qiagen). After 16–20 h post transfection, cells were reseeded at the density of 0.05 M cells per well of 96 well plate in DMEM high glucose containing 2% charcoal stripped serum. 4–6 h post seeding, PCCR-1 treatment was given for 24 h, followed by cells lysis with 1X Passive Lysis buffer of Dual luciferase kit (Promega). The signal was detected after addition of stop and glo reagent of the kit in luminescence mode.

### Translational studies: COPD Patient samples

Ethical approval for this ex vivo study from COPD patient sample was obtained from the Ethics Committee of Ethicos under independent Ethics committee held on Dec 30 2016 under the chairmanship of Dr Shubha Thatte for the study protocol no. GM/DP/2016/01 entitled ‘In vitro evaluation of Anti-inflammatory effect of novel chemical entities on tissue samples from COPD patients’. All translational studies and procedures were performed accordance with guidelines and regulations of the translational studies protocol no. GM/DP/2016/01.

Whole blood and BAL samples from COPD patients were obtained from Multispecialty hospital, Mumbai post approval from ethics committee and informed consent of patients. 30–40 mL Broncho alveolar lavage (BALf) was collected per patient and stored in ice. Blood was collected in heparin vaccutainer as per approved protocol. Both the samples were processed within 2 h of collection. BALF was passed through 100-µm and 40-µm cell strainer to remove debris and mucus, the samples were centrifuged and the cells were re-suspended in RPMI 1640 complete medium. PBMCs from blood were isolated using Histopaque-1077 by density gradient centrifugation. Isolated BAL cells and PBMCs were seeded in 96 well coated with 10 µg/ml anti-CD3 and 2 µg/ml anti-CD2. Cells were further treated with different concentration of test compound for 60 min followed by stimulation with 20 µg/ml anti-CD28 and incubated for 48–72 h. The supernatant was used for estimating IL-17 by ELISA.

### Animal study

Male C57BL/6 mice aged 8–10 weeks were obtained from the animal facility of Glenmark Pharmaceuticals Ltd. All animal experiments were approved by IAEC (institutional Animal Ethics Committee) as per the guideline set up by The Committee for the Purpose of Control and Supervision of Experiments on Animals (CPCSEA), India (CPCSEA is a statutory Committee of Department of Animal Husbandry and Dairying (DAHD), Ministry of Fisheries, Animal Husbandry and Dairying (MoFAH&D), India and constituted under the Prevention of Cruelty to Animals (PCA) Act, 1960., India). All animal experiments and procedures were performed in accordance with CPCSEA and IAEC guidelines and regulations (Registration number 231/2000/CPCSEA).In addition to that, all animal experimentation complies with the guidelines of Animal Research: Reporting In Vivo Experiments (ARRIVE). Male C57BL/6 mice were exposed to either room air or cigarette smoke (CS) from 10 cigarettes (Kentucky Research Cigarettes 3R4F) for 50 min, twice daily for 7 days in a whole body box exposure system (SIU24, ProMech Lab Holding AB, Sweden). PCCR-1 and Roflumilast were sonicated in phosphate buffered saline (PBS) containing 0.005% tween 80 for 20 min. The animals were administered vehicle or test compounds intranasally (i.n.) 40 µl (µl) per animal under isoflurane anesthesia. Cigarette smoke exposed mice were dosed intranasal either with vehicle or PCCR-1 (1, 10, 30, 50 and 100 µg per animal. n = 7 per group) once daily or Roflumilast 100 µg twice daily 1 h before the cigarette smoke exposure. Control animals were given vehicle one hour before exposure to fresh air or cigarette smoke. PCCR-1 and Roflumilast were sonicated in phosphate buffered saline (PBS) containing 0.005% tween 80 for 20 min. For localized delivery to lung, animals were administered vehicle or test compounds intranasal (i.n.) 40 µl (µl) per animal under isoflurane anesthesia. Animals were sacrificed 20 h after the last smoke exposure and broncho alveolar lavage fluid (BALf) was collected after euthanizing with a urethane, trachea was exposed and BALf was collected 4 times using 0.3 ml PBS. All aspirates of BAL were pooled and total number of cells were determined using a hemocytometer. BALf was centrifuged in cold and cell pellet was used for preparation of smears. BALf smear slides were stained with Leishmans stain and differential cell count of 500 cells based on standard morphology was performed manually. All data are presented as mean ± S.E.M. of animals.

### Ex vivo BAL studies

C57BL/6 mice were exposed to as per above mentioned protocol. On 7th day, animals were sacrificed and a tracheal cannula was inserted. Three times via the trachea, 1 mL of plain RPMI medium was instilled and recovered by gentle manual aspiration. The recovered BALf was centrifuged, the cell pellet washed twice and finally re-suspended in 5% FBS- RPMI. Isolated BAL cells were pre-incubated with different concentration of PCCR-1 for 60 min, followed by stimulating with 1 µg/ml soluble mouse anti-CD3 and 3 µg/ml mouse anti-CD28 mAbs and/or 100 ng/ml of LPS and incubated for 24-48 h at 37 °C. The supernatant was used for estimating IL-17 by ELISA.

### Pharmacokinetic (PK) studies in mice

A single dose intranasal PK study of PCCR-1 was conducted in male C57 mice at 20 and 50 µg/animal dose. The formulation was prepared in saline with 0.1% v/v tween-80 as the wetting agent. Animals were anesthetized briefly using gaseous isoflurane and oxygen mixture and a 40 µL of the formulation was instilled into both the nostrils (20 µL each) once the animal reached a regular breathing pattern and at pre-determined time points (0.25 h, 0.5 h, 1 h, 2 h, 4 h, 8 h and 24 h), approximately 0.3 mL of blood samples were collected using retro-orbital puncture in suitable anticoagulant (K3 EDTA). The animals were immediately sacrificed and lung was isolated. Blood samples were centrifuged (3000 rpm, 5 min) and plasma was harvested. Lung samples were homogenized in PBS buffer (pH 7.4) and centrifuged to collect the supernatant. Both plasma and lung samples were extracted using methyl t-butyl ether, mixed and centrifuged. The supernatant samples obtained after centrifugation were dried and reconstituted with acetonitrile-ammonium acetate buffer (90:10). The samples were mixed and analyzed in LCMS/MS for the analyte concentrations. Concentration vs time values were subjected to non-compartmental analysis using Phoenix WinNonlin® (Version 6.4) to estimate the appropriate pharmacokinetics parameters.

Additionally, after 7-day intranasal treatment with PCCR-1 in the CS-model in mice as described above, blood, BAL fluid and lung tissues were collected on day-8 approximately 1 h after the last treatment dose for estimating the drug concentrations for PK-PD correlation.

### Toxicology studies

The 14-day dose-range inhalation toxicology study was conducted at Charles River Laboratories Edinburgh Ltd, Elphinstone Research Centre, Tranent, East Lothian, EH33 2NE, UK, under the Home Office Project License No. PPL 70/8778, Toxicology of Pharmaceuticals. The UK Home Office controls scientific procedures on animals in the UK and does so by the issue of licences under the Animals (Scientific Procedures) Act 1986. The regulations conform to EU Directive 2010/63/EU and achieve the standard of care required by the US Department of Health and Human Services' Guide for the Care and Use of Laboratory Animals.

The toxicology study was done in SD (Sprague Dawley) rats that were exposed to PCCR-1 aerosol for 60 min daily at 3.4, 10.8 and 32.1 mg/kg/day (delivered dose) for 14 days using a snout only exposure technique via a modular stainless steel flow past inhalation chamber. A 60% w/w blend of PCCR-1 in Lactose LH201 was prepared by adding the appropriate amount of PCCR-1 to the appropriate amount of lactose. The estimated delivered doses were derived based on analytical aerosol concentration, and respiratory minute volume calculated based on body weight, exposure duration, and animal body weight (Supplementary section B).

### Statistical analysis

Statistical differences between groups were analyzed by one-way analysis of variance (ANOVA) followed by Dunnett’s multiple comparison test using Graph Pad Prism (version-5.01, GraphPad Software Inc., CA). Graph Pad Prism software (version 5.01, GraphPad Software Inc., CA) was used for the generation of graphs. Statistical difference between two groups were determined by pared t-test and p value < 0.05 were considered statistical significant.

## Supplementary Information

Supplementary Information.

## References

[1] Agusti, A.G & Vogulmeier, C. Gold 2020. https://goldcopd.org/wp-content/uploads/2019/12/GOLD-2020-FINAL-ver1.2-03Dec19_WMV.pdf

[2] Singh D (2015). Chronic obstructive pulmonary disease, neutrophils and bacterial infection: A complex web involving IL-17 and IL-22 unravels. E Bio Med..

[3] Barjaktarevic IC (2015). Supplemental oxygen therapy for patients with chronic obstructive pulmonary disease. Semin. Respir. Crit. Care Med..

[4] Watz H (2014). Efficacy and safety of the p38 MAPK inhibitor losmapimod for patients with chronic obstructive pulmonary disease: A randomised, double-blind, placebo-controlled trial. Lancet Respir. Med..

[5] Begg M (2021). Exploring PI3Kδ molecular pathways in S13 COPD and following an acute exacerbation, two randomized controlled trials. Int. J. Chron. Obstruct. Pulmon. Dis..

[6] Baye, J. Roflumilast (daliresp): A novel phosphodiesterase-4 inhibitor for the treatment of severe chronic obstructive pulmonary disease*. J. Formul. Manag*, **37**, 149–16 (2012) http://www.ncbi.nlm.nih.gov/pmc/articles/pmc3351880/PMC3351880 22605906

[7] Gupta S (2012). Side-effects of roflumilast. Lancet.

[8] Barnes PJ (2008). Emerging pharmacotherapies for COPD. Chest.

[9] Page CP, Spina D (2012). Selective PDE inhibitors as novel treatments for respiratory diseases. Curr. Opin. Pharmacol..

[10] Pinner NA, Hamilton LA, Hughes A (2012). Roflumilast: A phosphodiesterase-4 inhibitor for the treatment of severe chronic obstructive pulmonary disease. Clin. Ther..

[11] Le Rouzic O, Roche N, Cortot AB (2018). Defining the ‘frequent exacerbator’ phenotype in COPD: A hypothesis-free approach. Chest.

[12] Barnes PJ (2013). Corticosteroid resistance in patients with asthma and chronic obstructive pulmonary disease. J. Allergy Clin. Immunol..

[13] Xue X (2021). Preclinical and clinical characterization of the RORγt inhibitor JNJ-61803534. Sci. Rep..

[14] Liu S (2021). Discovery of a novel RORγ antagonist with skin-restricted exposure for topical treatment of mild to moderate psoriasis. Sci. Rep..

[15] Fukuzaki S (2021). Preventive and therapeutic effect of anti-IL-17 in an experimental model of elastase-induced lung injury in C57Bl6 mice. Am. J. Physiol. Cell Physiol..

[16] Chung KF (2001). Cytokines in chronic obstructive pulmonary disease. Eur. Respir. J..

[17] Chang Y, Nadigel J, Boulais N, Bourbeau J, Maltais F, Eidelman DH, Hamid O (2011). CD8 positive T cells express IL-17 in patients with chronic obstructive pulmonary disease. Respir. Res..

[18] Duan M, Tang H, Zhong X, Huang Y (2013). Persistence of Th17/Tc17 Cell expression upon smoking cessation in mice with cigarette smoke-induced emphysema. Clin. Dev. Immunol..

[19] Roos AR, Sande C, Mori M, Bjermer L, Martin R, Stampfli MR, Erjefa JS (2015). IL-17A is elevated in end-stage chronic obstructive pulmonary disease and contributes to cigarette smoke–induced lymphoid neogenesis. Am. J. Respir. Crit. Care Med..

[20] Grove KCD, Provoost S, Verhamme FM, Bracke KR, Joos GF, Maes T, Brusselle GG (2016). Characterization and quantification of innate lymphoid cell subsets in human lung. PLoS ONE.

[21] Wang X (2012). Transcription of IL-17 and IL-17f is controlled by conserved noncoding sequence 2. Immunity.

[22] Shen N, Wang J, Zhao M, Pei F, He B (2011). Anti-interleukin-17 antibodies attenuate airway inflammation in tobacco-smoke-exposed mice. Inhal. Toxicol. Int. Respir. Res..

[23] Vlahos R, Bozinovskiab S (2015). Preclinical murine models of chronic obstructive pulmonary disease. Eur. J. Pharmacol..

[24] Christenson SA (2019). An airway epithelial IL-17A response signature identifies a steroid-unresponsive COPD patient subgroup. Clin. Invest..

[25] Dong L (2014). Effect of lianhuaqingwen capsules on airway inflammation in patients with acute exacerbation of chronic obstructive pulmonary disease. Evid.-Based Complement. Alternat. Med..

[26] Maneechotesuwan K, Kasetsinsombat K, Wongkajornsilp A, Barnes PJ (2013). Decreased indoleamine 2, 3-dioxygenase activity and IL-10/IL-17A ratio in patients with COPD. Thorax.

[27] Zhang L, Cheng Z, Liu W, Wu K (2013). Expression of interleukin (IL)-10, IL-17A and IL-22 in serum and sputum of stable chronic obstructive pulmonary disease patients. COPD.

[28] Chu S, Zhong X, Zhang J, Lao Q, He Z, Bai J (2011). The expression of Foxp3 and ROR gamma t in lung tissues from normal smokers and chronic obstructive pulmonary disease patients. Int. Immunopharmacol..

[29] Chaudhari S.S., Thomas, A., Kadam A.B., Dhone S.V., Adik B.G., Joshi N.K., Shah D.M., Bajpai M. Aryl and heteroaryl ether compounds as ROR gamma modulators. WO2015159233A1

[30] Anton MJ (2009). Retinoid-related orphan receptors (RORs): Critical roles in development, immunity, circadian rhythm, and cellular metabolism. NRS.

[31] Rouzic OL (2017). Th17 cytokines: novel potential therapeutic targets for COPD pathogenesis and exacerbations. Eur. Respir. J..

[32] Doe C (2010). Expression of the T helper 17-associated cytokines IL-17A and IL-17F in asthma and COPD. Chest.

[33] Cazzola M, Matera MG (2012). IL-17 in chronic obstructive pulmonary disease. Expert Rev. Respir. Med..

[34] Kuwabara T, Ishikawa F, Kondo M, Kakiuchi T (2017). The role of IL-17 and related cytokines in inflammatory autoimmune diseases. Mediat. Inflamm..

[35] Andelid K (2015). Systemic cytokine signaling via IL-17 in smokers with obstructive pulmonary disease: A link to bacterial colonization?. Int. J. COPD.

[36] Zhang X (2011). Increased interleukin (IL)-8 and decreased IL-17 production in chronic obstructive pulmonary disease (COPD) provoked by cigarette smoke. Cytokine.

[37] Dominique MA, Bullens A, Ann D, Sven S, Lieven J (2013). Dupont IL-17A in human respiratory diseases: Innate or adaptive immunity? Clinical implications. Clin. Dev. Immunol..

[38] Stefano AD (2009). T helper type 17-related cytokine expression is increased in the bronchial mucosa of stable chronic obstructive pulmonary disease patients. Clin. Exp. Immunol..

[39] Pridgeon C, Bugeon L, Donnelly L, Straschil U, Tudhope SJ, Fenwick P, Lamb JR, Barnes PJ, Margaret J (2011). Regulation of IL-17 in chronic inflammation in the human lung. Clin. Sci..

[40] Lu Y (2019). Effectiveness of long-term using statins in COPD—a network meta-analysis. Respir. Res..

[41] Morales JMGR (2020). Critical role of interleukin (IL)-17 in inflammatory and immune disorders: An updated review of the evidence focusing in controversies. Autoimmun. Rev..

[42] Eustace A (2011). Identification of cells expressing IL-17A and IL-17F in the lungs of patients with COPD. Chest.

[43] Wu Q (2013). RORct modulates macrophage recruitment during a hydrocarbon oil-induced inflammation. PLoS ONE.

[44] Wu Q (2013). RORγt modulates macrophage recruitment during a hydrocarbon oil-induced Inflammation. PLoS ONE.

[45] Aul R (2012). Inhaled LPS challenges in smokers: A study of pulmonary and systemic effects. Br. J. Clin. Pharmacol..

[46] Gupta V (2014). Characterization of the inflammatory response to inhaled lipopolysaccharide in mild to moderate chronic obstructive pulmonary disease. Br. J. Clin. Pharmacol..

[47] Disse B, Speck GA, Rominger KL, Witek TJ, Hammer R (1999). Tiotropium (Spiriva): Mechanistical considerations and clinical profile in obstructive lung disease. Life Sci..

[48] Tashkin DP (2005). Is a long-acting inhaled bronchodilator the first agent to use in stable chronic obstructive pulmonary disease?. Curr. Opin. Pulm. Med..

[49] Tamura G, Ohta K (2007). Adherence to treatment by patients with asthma or COPD: Comparison between inhaled drugs and transdermal patch. Respir. Med..

[50] Casarosa P (2009). Preclinical evaluation of long-acting muscarinic antagonists: Comparison of tiotropium and investigational drugs. J. Pharma. Expert. Ther..

[51] Zein RME (2019). Retinoic acid receptor α as a novel contributor to adrenal cortex structure and function through interactions with Wnt and Vegfα signaling. Sci. Rep..

[52] Rosol TJ, Yarrington JT, Latendresse J, Capen CC (2001). Adrenal gland: Structure, function, and mechanisms of toxicity. Toxicol. Pathol..

[53] Everds NE, Snyder PW, Bailey KL, Bolon B, Creasy DM, Foley GL, Rosol TJ, Sellers T (2013). Interpreting stress responses during routine toxicity studies: A review of the biology, impact, and assessment. Toxicol. Pathol..

[54] Harvey, P.W., Everret, D.J., Springall, CJ. Adrenal Toxicology: Molecular Targets, Endocrine Mechanisms, Hormonal Interactions, Assessment Models, and Species Differences in Toxicity. In, Target Organ Toxicology Series, 26. AW Hayes, JA Thomas, DE. Boca Raton: CRC Press, pp 1–35. Gardner (series eds 2008)

[55] Harvey PW, Sutcliffe C (2010). Adrenocortical hypertrophy: Establishing cause and toxicological significance. J. Appl. Toxicol..

[56] Sievers F (2011). Fast, scalable generation of high-quality protein multiple sequence alignments using Clustal Omega. Mol. Syst. Biol..

