# Supplementary material for: An inhibitor of RORγ for chronic pulmonary obstructive disease treatment
Source: Sci Rep. 2022 May 24;12:8744. doi: 10.1038/s41598-022-12251-z (PMC9130233; doi:10.1038/s41598-022-12251-z)
Supplement: Supplementary file 1 — Supplementary Information. [file 41598_2022_12251_MOESM1_ESM.docx]

**An inhibitor of RORγ for chronic pulmonary obstructive disease treatment**

Harshada Desai^1*^, Megha Marathe^1*^, Varada Potdar^1*^, Prabhakar Tiwari^1*^, Ashwini Joshi^1*^; Sheetal R Kadam^1^, Arti Rajesh Joshi^1^, Abhay Kulkarni^1^, Vikram Bhosale^1^, Avinash Hadambar^1^, Bhavik Lodhiya^1^, Venkatesha Udupa^2^, Dayanidhi Behera^3^, Sachin S. Chaudhari^4^, Sanjib Das^4^, Malini Bajpai^1 #^**¶.**  Nagaraj Gowda^1,3#^, Pravin S Iyer^5#^

**^1^Department of Biological Research, ^2^Department of Toxicology, ^3^Department of Drug Metabolism and Pharmacokinetics, ^4^Department of Chemical Research and ^5^Head NCE Research, Glenmark Pharmaceuticals Limited, Glenmark Research Centre A-607, MIDC Mahape, Navi Mumbai 400709. India.**

**¶Correspondence:**

**Dr. Malini Bajpai**

**Glenmark Research center, Navi Mumbai**

**Email: malini.bajpai@glenmarkpharma.com**

***Contributed equally**

**# Contributed equally**

**Supplementary Figure 1. IL-17 and IL-17F inhibition study in rat spleenocyte and human PBMC**

**B.**

**A.**

**C..**

**Supplementary Figure 1a**. Dose dependent inhibition of IL-17 cytokine from rat splenocytes.

**Supplementary Figure 1b.** Dose dependent inhibition of IL-17F cytokine from human PBMC.

**Supplementary** Figure 1c. Expression RORγt mRNA and IL-17 mRNA in PCCR-1 treated PBMC (n=1). Effect of PCCR-1 on RORγt and IL-17 gene expression in PCCR-1 treated PBMCs. Data were analyzed using the one-way ANOVA test. (*, ***) indicate P values of less than 0.05 and 0.001 respectively.

**Supplementary Figure 2. IL-17 inhibition across mouse and human neutrophils and mouse macrophages and RORγ transactivation activity**

**B.**

**A.**

**D.**

**C.**

Supplementary Figure 2. Dose dependent inhibition of IL-17 in (a) mouse neutrophils (n=2), (b) mouse macrophages (n=2) and (c) human neutrophils (n=4). (d) dose dependent inhibition of RORγ transactivation in human RORγ transfected HEK293 cells (n=4). ‘n’ is the representative of number of experimental replicates.

**Supplementary Figure 3. PK-PD correlation with PCCR-1 in CS model in mice**


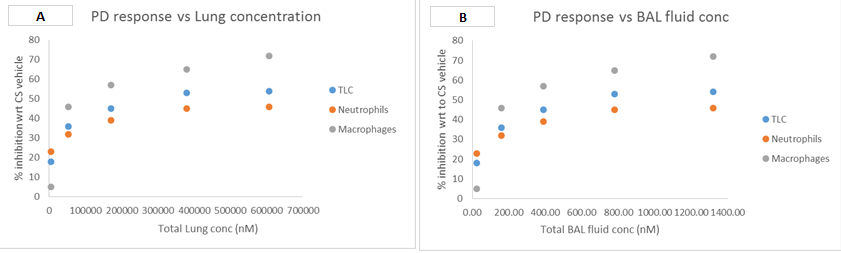


**Supplementary Figure 3.** PK-PD correlation with PCCR-1 in CS model in mice. Good correlation of the lung (A) and BAL fluid (B) concentrations versus inhibition of the PD biomarkers (total leukocyte count, neutrophils and macrophages) were observed with good dose-dependent inhibition profiles

**Supplementary Table 1.** Pharmacokinetic parameters of PCCR-1 after intranasal dose in male C57 mice


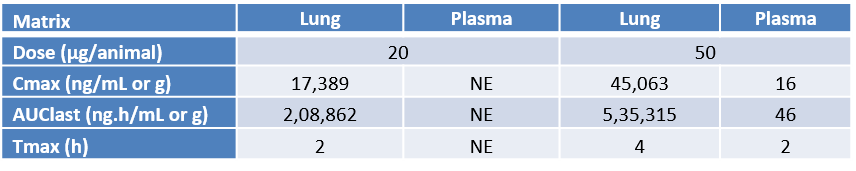


**Supplementary Table 1**. Pharmacokinetic parameters of PCCR-1 after intranasal dose in male C57 mice. NE means no measurable concentrations observed (below limit of quantification)
